# Assessment of the knowledge and behavior of backyard and small-scale producers in California regarding disease prevention, biosecurity practices and antibiotics use

**DOI:** 10.1371/journal.pone.0277897

**Published:** 2022-11-21

**Authors:** Kyuyoung Lee, Richard V. Pereira, Beatriz Martínez-López, Roselle C. Busch, Alda F. A. Pires

**Affiliations:** 1 Department of Medicine and Epidemiology, Center for Animal Disease Modeling and Surveillance, School of Veterinary Medicine, University of California-Davis, Davis, California, United States of America; 2 Department of Population Health and Reproduction, School of Veterinary Medicine, University of California-Davis, Davis, California, United States of America; University of Illinois Urbana-Champaign College of Veterinary Medicine, UNITED STATES

## Abstract

The number and popularity of backyard poultry and livestock farming have rapidly increased in California as well as other states in the United States following consumers’ preference for local and organic products in the last few years. This study aimed to investigate current on-farm management and farmers’ understanding of Veterinary Feed Directive (VFD) and California Senate Bill (SB) 27 implications for disease prevention, biosecurity procedures, and antimicrobial use in small-scale and backyard farms in California. The survey consisted of 38 questions. The responses of 242 backyard and small-scale livestock owners were investigated in this study. Descriptive statistics summarized survey responses, and multivariable logistic regression evaluated the association of antibiotics purchase and use, and the impact of VFD and SB27 on antibiotic use with demographics and on-farm management. Backyard and small-scale farmers in California mostly raised chickens or small ruminants with small herd sizes kept for personal use. Antibiotics were generally used for individual treatment of a sick animal with the guidance of a veterinarian. VFD and SB27 implementation promoted the judicious use of antibiotics, specifically, by enhancing the relationship between backyard and small-scale farmers with veterinarians and treating fewer animals with antibiotics under veterinary oversight. Therefore, better access to veterinary service in backyard and small-scale farms will improve the farmer’s knowledge of good husbandry practices with judicious antimicrobial use in livestock and finally contribute to reducing the risk of antimicrobial resistance in California.

## Introduction

The number of backyard poultry and livestock owners has exponentially increased in California and other states in the U.S over the past two decades [1–4]. The high popularity of backyard poultry and livestock is possibly related to consumers’ increasing interest in local food production, food sustainability, and preference for fresh, local, and organic products [5]. Backyard chicken is the most popular animal raised on backyard farms because of the ease of keeping chickens and the benefit of table eggs [6, 7]. Goat and sheep have also become popular backyard livestock as pets and sources of animal products (eggs, meat, milk, and fiber) [8, 9]. The rapid growth of peridomestic or backyard livestock and poultry “farming” in urban and peri-urban areas poses challenges associated with disease control in backyard premises due to the lack of access to adequate veterinary care for technical information and knowledge of disease prevention and biosecurity procedures [4]. Moreover, infrequent or lack of veterinary oversight may lead to inappropriate treatments and poor animal health and welfare [10, 11]. In a recent survey in four US western states, only 43% of small-scale and backyard livestock owners sought veterinary care in the past year, with concerns for infectious and parasitic diseases as the most common reasons. The access to veterinary care showed differences by their location (e.g., state and urban or peri-urban setting) and species owned [4]. As a consequence, the sources of information about disease prevention and biosecurity procedures and how to perform treatment and procedures, sought by small-scale and backyard livestock owners have mainly been from the internet (81.8% and 70.7%, respectively) rather than veterinarians (61.5% and 58.0%, respectively) [4, 10].

The lack of access to technical information and proper diagnosis through veterinary oversight for backyard and small-scale farmers may increase the risk of zoonotic disease, introduction of foreign animal disease outbreaks to commercial livestock farms (e.g., virulent Newcastle Disease and Highly Pathogenic Avian Influenza), as well as non-compliance with drug withdrawal intervals, drug residues, and the potential of antimicrobial resistance (AMR) development through misuse of antimicrobial and prohibited substances in livestock species [2, 12–18]. New zoning regulations implemented in several cities in California (e.g., Los Angeles, San Diego, and Sacramento) allow poultry and goats in select residential areas [1, 2, 9, 11]. Moreover, recent federal and state requirements for veterinary oversight of in-feed/water antibiotics (Veterinary Feed Directive, VFD) [19] and requirements of prescription for common over-the-counter antibiotics in California (SB27) [20] have increased the need for small-scale livestock producers to develop Veterinary-Client-Patient-Relationships (VCPR) for obtaining access to antibiotics that otherwise may have been obtained over-the-counter.

Therefore, we performed a cross-sectional study to evaluate current farm management practices and the understanding of disease prevention and biosecurity procedures, the use of antibiotics, and new regulations (e.g., VFD and SB27) on small-scale and backyard farms in California. Our study provides an insight into farm management and antibiotic use that can lead to better husbandry practices with judicious antimicrobial use in livestock and poultry on Californian backyard and small-scale farms.

## Materials and methods

### Study population and survey instrument

The semi-structured survey consisted of 38 questions, including a combination of binary, categorical, and open-ended questions, and was conducted using an online survey platform as well as hard-copy surveys during in-person interviews [S1 Table]. The survey targeted backyard and small-scale livestock and poultry owners, and investigated disease prevention and biosecurity procedures including general demographics, current antibiotic purchase and use, veterinary services, VCPR, and the impact of new regulations (VFD and SB27) on their antibiotic use. We recruited the five members of the faculty and staff at the University of California, Davis who had expertise in backyard farming and closely worked with farmers, to pre-test the survey tool and perform the final review for better validity and reliability of the questionnaire. The survey instrument was reviewed by the University of California, Davis Institutional Review Board, and was granted exemption approval (IRB Number # 1316273–1). The survey was introduced at five workshops on animal health and antibiotic use in backyard livestock and poultry organized by the research team (Sonoma, Stanislaus, Contra Costa, Santa Clara, and Kern counties, CA, between September 2018 and March 2019). Hard copies of the survey were available at these workshops during the study period. The online survey was advertised on social media (e.g., Facebook, Twitter), in newsletters, and on listservs of Cooperative Extension County offices, local and regional small-scale holder interest group websites, and by word-of-mouth. The cover letter described the selection criteria for backyard poultry and livestock animals (poultry, cattle, swine, sheep, goats, or camelids) and small-scale farm/premise, and provided a brief explanation of federal and state regulations regarding antimicrobial use. In addition, the cover letter included consent language, and the research team and IRB contacts. The online survey was available to respondents between January 23, 2019 and September 30, 2019.

### Data management and statistical analysis

The data collected from the survey was anonymous for confidentiality, and any personal information of respondents was not retained. Only surveys that completely answered key questions (demographics, on-farm management of antibiotics in a premise, and perception of VFD and SB27 on antibiotics use) were included in the final analysis [S1 Table]. The answers to the survey were converted into binary, multinomial, ordinal, and categorical variables [S2 Table]. Descriptive statistics were used to summarize survey results with frequency tables and graphs. Multiple-choice binary responses of general demographics with high similarity were merged into one category due to overlap or synonymous response meaning: (1) The question about the reasons for raising livestock: “pet,” “hobby,” and “rescue” into “pet/hobby/rescue”; “backyard producer for sale of live animal” and “backyard producer for sale of animal products” into “backyard producer for sale”; “small-scale farmer (livestock only)” and “small-scale farmer (vegetable and livestock)” into “small-scale farmer”; (2) The question about species of livestock/poultry: “duck,” “geese,” and “turkey” into “duck/geese/turkey”; “goats,” “sheep,” “llama,” and “alpaca” into “small ruminants/camelid”; “beef cattle” and “dairy cattle” into “cattle.” In addition, responses about herd/flock size were re-categorized from six categories (1–5, 6–10, 11–20, 21–50, 51–100, and >100) to four categories (1–10, 11–20, 21–50, and > 50), considering the herd/flock size distribution of respondents’ farms. Other open-ended responses were manually reviewed and re-categorized into new categories [S1 Table].

We used multiple methods for the analysis. Factor analysis of mixed data (FAMD) and hierarchical clustering analysis were used to identify clusters of backyard and small-scale livestock owners considering demographic factors: the location of property, the reasons for raising livestock, herd/flock size, and livestock/poultry species on a farm. Multivariable logistic regression was performed to evaluate the association of four key outcome binary variables: (1) the purchase of antibiotics and (2) the use of antibiotics in the past year prior to the survey, the impact of (3) VFD, and (4) SB27 legislation on their antibiotic use in the past year prior to the survey, with farm demographics and management factors (e.g., antimicrobial use, veterinary care, treated diseases, VCPR). The model selection was conducted by backward stepwise selection based on Akaike information criteria comparison. All descriptive statistics, FAMD, hierarchical clustering analysis, logistic regression, and graphic representation were conducted in R studio (Version 4. 1. 2, R Core Team) [21]. FAMD and hierarchical clustering analysis were specifically performed using Factoextra package [22]. A *P* value of ≤ 0.05 was considered a significant difference.

## Results

### General demographics

**Fig 1:**
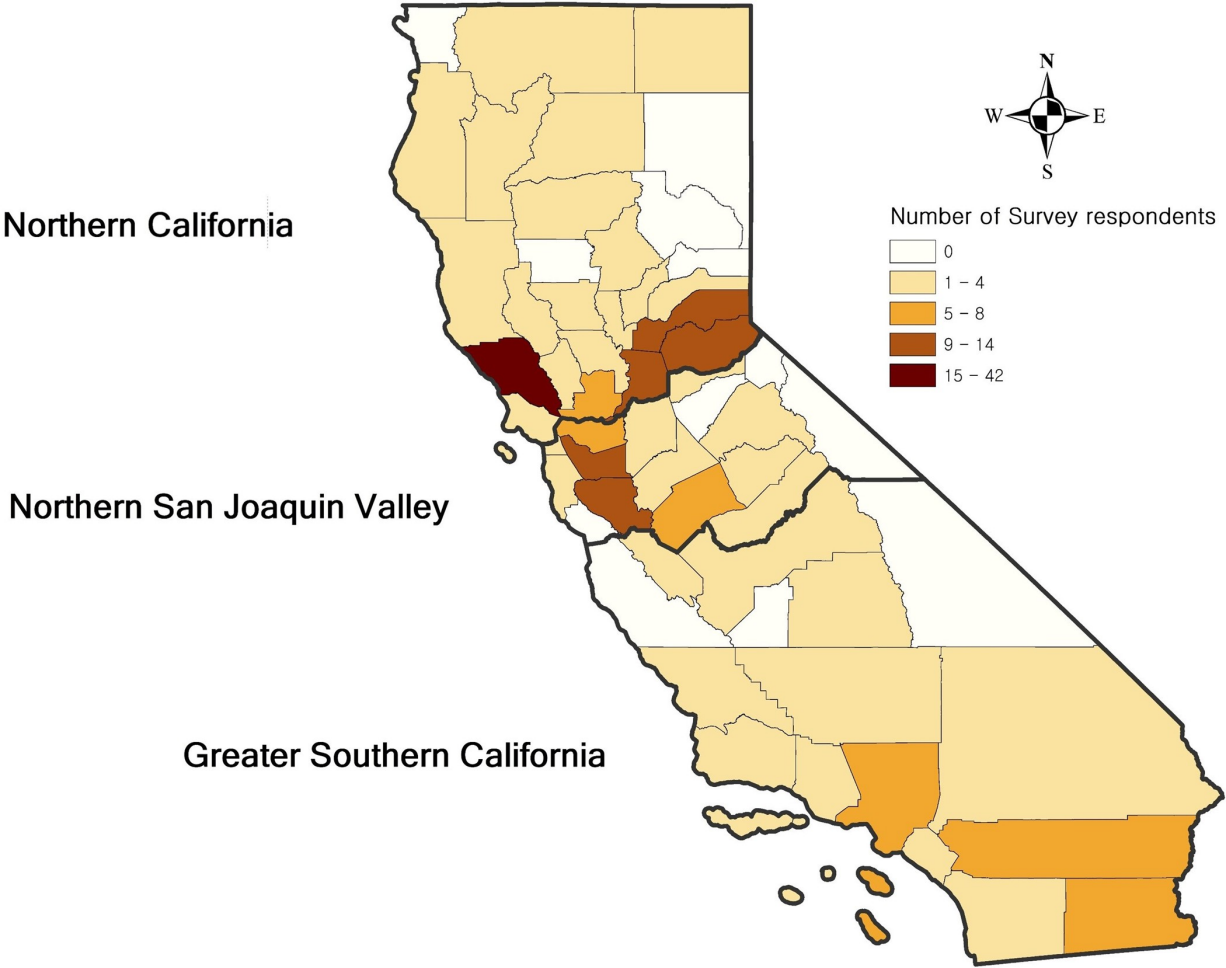
Geographical distribution of respondents’ premises by counties in California (n = 242).

The present study included 242 participants (202 online and 40 hardcopy responses) who completely answered key questions, and excluded 49 respondents among 293 participants in this study (Response rate: 82.6%, 242/293). All respondents resided in California, and their premises were located in 46 counties (No answer: 12.4%). Sonoma County included the largest number of respondents (19.2%, 42/253), and other counties had from one to twelve respondents each [Fig 1].

Participant premises were mostly located in rural areas (54.5%) [Table 1]. Respondents mostly raised livestock/poultry for personal use (67.4%), but around one-third of respondents sold their livestock and/or livestock products. Around half of respondents raised livestock/poultry as pets/hobby/rescue (54.1%) and/or for breeding purposes (45.9%). Around 40% of respondents were either 4-H or FFA members (see Table 1 for definition). Most respondents raised chickens (82.6%) and/or small ruminants (56.6%).

**Table 1:** Summary of farm demographics of the survey respondents (n = 242).

Demographic characteristics	Number	Proportion (%)
**Location of farms**		
⬩ Rural	132	54.5
⬩ Suburban	47	19.4
⬩ Urban	31	12.8
⬩ Town	31	12.8
⬩ Unknown	1	0.4
**Reason to raise livestock/poultry** ^**#**^		
⬩ Backyard producer for personal use	163	67.4
⬩ Pets/Hobby/Rescue	131	54.1
⬩ Breeder	111	45.9
⬩ 4-H* or FFA** member	99	40.9
⬩ Backyard producer for sale	80	33.1
⬩ Small-scale famers	68	28.1
⬩ Other***	5	2.1
**Species of livestock/poultry** ^**#**^		
⬩ Chicken	200	82.6
⬩ Small Ruminants (Goats/Sheep/Alpaca/Llama)	137	56.6
⬩ Duck/Geese/Turkey	69	28.5
⬩ Equids (Horse/Mules/Donkeys)	68	28.1
⬩ Swine	37	15.3
⬩ Cattle	34	14.0
⬩ Non-Livestock/Exotic pets****	53	21.9
**Number of Species in a farm**		
⬩ 1–2	134	55.4
⬩ 3–4	83	34.3
⬩ ≥5	23	9.5
⬩ Unknown	2	0.8

* 4-H is a US youth program housed by land-grant universities’ Cooperative Extension. This program provides developmental opportunities to youth (age 5 to 19) in rural farming communities, urban neighborhoods, and schoolyards. Skills learned include agriculture, animal husbandry, community service, and personal development

** FFA: National FFA Organization (formerly Future Farmers of America) is a US intracurricular student organization. It gives students experience in agriculture and leadership (middle and high school classes).

*** Other reason to raise livestock/poultry: School Garden, Packing, Fairground & Experimental use

**** Non-Livestock/Exotic pets: Rabbit, Dog, Bee, Cavy, Turtle, Emu, Guinea fowls, Peacock, Quail

^#^ This question allowed respondent to select multiple responses

**Fig 2:**
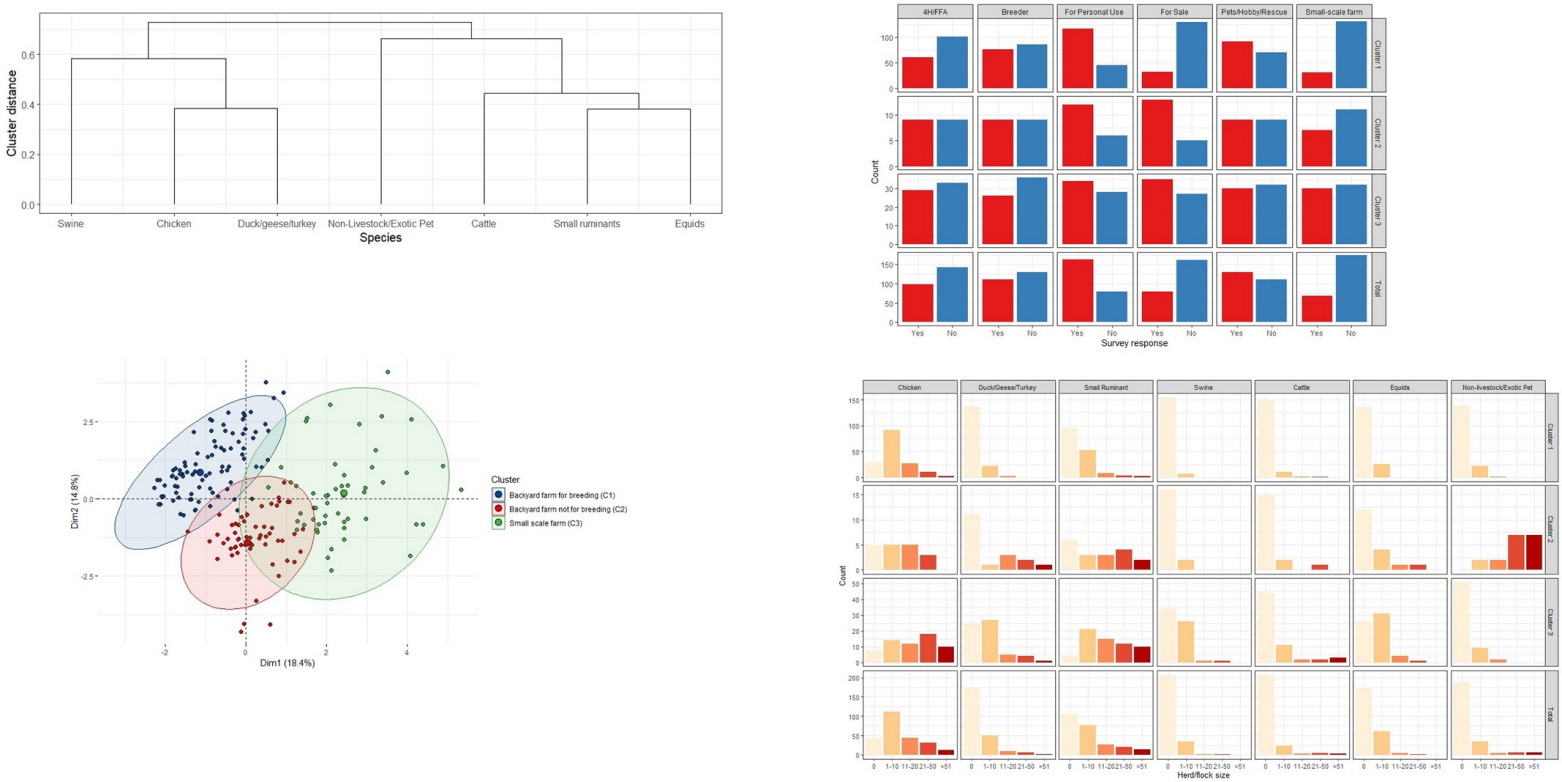
Summary of respondents’ farm demographics and clustering analysis. (A) Dendrogram of respondents’ tendency to raise livestock/poultry species (B) Distribution of three clusters identified by hierarchical clustering analysis combining factor analysis of mixed data (C) The respondents’ reason to raise livestock/poultry in the three clusters (D) Species and herd/flock size of livestock/poultry in the three clusters. * 4-H is a US youth program housed by land-grant universities’ Cooperative Extension. This program provides developmental opportunities to youth (age 5 to 19) in rural farming communities, urban neighborhoods, and schoolyards. Skills learned include agriculture, animal husbandry, community service and personal development. FFA: National FFA Organization (formerly Future Farmers of America) is a US intracurricular student organization. It gives students experience in agriculture and leadership (middle and high school classes).

About half of respondents raised one or two species [Table 1]. The remaining respondents raised three or more species and they tended to raise similar species of animals, either poultry (chicken and ducks/geese/turkey) or livestock (small ruminants, equids, and cattle) [Fig 2A]. Hierarchical clustering analysis found three clusters of respondents based on their farm demographics [Fig 2B]. Cluster 1 involved backyard farmers raising a small number of chickens and/or small ruminants/camelids for breeding and/or pet/hobby/rescue (47.5%, 115/242). Cluster 2 included backyard farmers raising a small number of chickens and small ruminants mostly for personal use, but not for breeding (31.0%, 75/242). Cluster 3 involved small-scale farmers raising livestock and/or poultry with larger herd/flock sizes for sale (21.5%, 52/242). More than half of small-scale farmers (Cluster 3 = 53.9%) sold their livestock and/or livestock products; this proportion was higher compared to backyard farmers (Cluster 1 = 34.7%, Cluster 2 = 19.0%) [Fig 2C]. The herd/flock size of backyard farms (Clusters 1 and 2) was generally 1 to 10 heads, which were smaller than the small-scale farms (Cluster 3) (median flock size of chicken: 11 to 20; and herd size of small ruminants: 21 to 50) [Fig 2D].

### Antibiotic use in livestock/poultry and veterinary services

**Fig 3:**
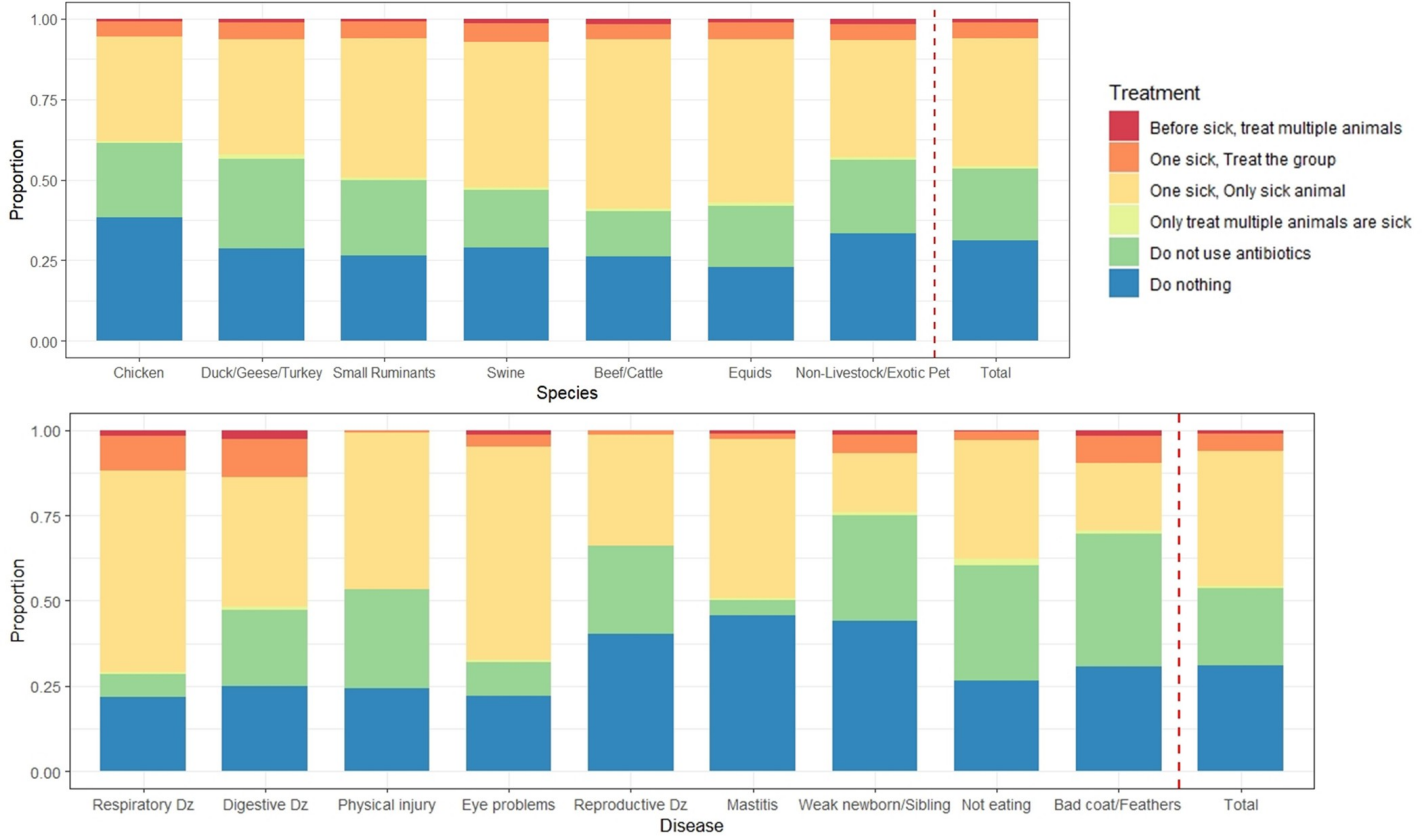
Summary of antibiotic treatment to livestock/poultry by type of diseases and species.

Around 40% of respondents purchased antibiotics for their livestock/poultry in the past year prior to the survey [Table 2]. More than half of small-scale farmers (Cluster 3) purchased antibiotics, which was greater than the proportion of backyard farmers (Clusters 1 and 2). The survey respondents mainly purchased antibiotics for their livestock/poultry from veterinarians (63.5%). Around 40% of respondents also reported that they used antibiotics in livestock/poultry in the past year prior to the survey. Around two-thirds of small-scale farmers used antibiotics in livestock/poultry, which was greater than the proportion of backyard farmers. Only a few respondents administered antibiotics in water (5.8%) and feed (12.0%). Respondents generally used antibiotics in livestock/poultry following a veterinarian prescription (72.1%), but around half of the respondents reported that they used antibiotics based on their own decision without a prescription. Around 40% of respondents used antibiotics for the individual treatment of a sick animal [Fig 3]. A smaller proportion of respondents treated for a sick animal, but not with antibiotics (22.5%) or did not treat them at all (31.1%). Respondents used antibiotics in equids or cattle more frequently than in poultry. They used antibiotics for treatment of respiratory and digestive diseases or eye problems more frequently than other diseases.

**Table 2:** Summary of antibiotics use of the survey respondents (n = 242).

Responses	Number	Proportion (%)
**Purchase antibiotics in the past year prior to the survey**		
⬩ No	146	60.3
⬩ Yes	96	39.7
✓ Cluster 1: Backyard farm for breeding (n = 115)	46	40.0
✓ Cluster 2: Backyard farm not for breeding (n = 75)	24	32.3
✓ Cluster 3: Small-scale farms (n = 52)	26	50.0
**Source of antibiotics purchase** ^**#**^		
⬩ Directly from a veterinarian	61	63.5
⬩ Directly from feed store/retail store	35	36.5
⬩ Online/internet	14	14.6
⬩ Delivered from a drug distributor with the order placed by a veterinarian	10	10.4
⬩ Directly from a drug distributor (ex: AHI)	4	4.2
**Use antibiotics in the past year prior to the survey**		
⬩ Unknown	4	1.7
⬩ No	136	56.2
⬩ Yes	102	42.1
✓ Cluster 1: Backyard farm for breeding (n = 115)	48	41.7
✓ Cluster 2: Backyard farm not for breeding (n = 75)	22	29.3
✓ Cluster 3: Small-scale farms (n = 52)	32	61.5
**Who made a decision of antibiotics use for livestock/poultry** ^**#**^		
⬩ Veterinarian	75	73.5
⬩ Owner of farm/backyard owner	54	52.9
⬩ Family member	10	9.8
⬩ Service manager	2	2.0
⬩ Employee	0	0
**Use antibiotics in water in the past year prior to the survey**		
⬩ Unknown	4	1.7
⬩ No	224	92.6
⬩ Yes	14	5.8
✓ Cluster 1: Backyard farm for breeding (n = 115)	7	6.1
✓ Cluster 2: Backyard farm not for breeding (n = 75)	5	6.7
✓ Cluster 3: Small-scale farms (n = 52)	2	3.8
**Use antibiotics in feed in the past year prior to the survey**		
⬩ Unknown	4	1.7
⬩ No	209	86.4
⬩ Yes	29	12.0
✓ Cluster 1: Backyard farm for breeding (n = 115)	12	10.4
✓ Cluster 2: Backyard farm not for breeding (n = 75)	9	12.0
✓ Cluster 3: Small-scale farms (n = 52)	8	15.4

# This question allowed respondent to select multiple responses

In the past year prior to the survey, around half of respondents used veterinary services [Table 3]. A higher proportion of small-scale farmers (Cluster 3 = 71.2%) used veterinary services compared to backyard farmers (Cluster 1 = 59.1%, Cluster 2 = 41.3%). In the past year, the most frequent veterinary service required by farmers was emergency calls (e.g., dystocia, multiple sick animals, sudden illness, etc.), followed by regular/routine services. Only one-third of respondents had a VCPR, with a higher proportion among small-scale farmers compared to backyard farmers.

**Table 3:** Summary of veterinary service use by survey respondents (n = 242).

Responses	Number	Proportion (%)
**Veterinary services in the past year prior to the survey**		
⬩ Unknown	24	9.9
⬩ No	82	33.9
⬩ Yes	136	56.2
✓ Cluster 1: Backyard farm for breeding (n = 115)	68	59.1
✓ Cluster 2: Backyard farm not for breeding (n = 75)	31	41.3
✓ Cluster 3: Small-scale farms (n = 52)	37	71.2
**Reason of veterinary service in the past year** ^**#**^		
⬩ Emergencies (ex: dystocia, multiple sick animals, sudden illness, etc.)	84	61.3
⬩ Regular or routine visits (ex: pregnancy checks, herd health visits, vaccinations, etc.)	81	59.1
⬩ Consulted over the phone or by email	71	51.8
⬩ For certificates of veterinary inspection (ex: health certificates)	26	19.0
⬩ For feed VFDs and water prescriptions	2	1.5
**Have a Veterinarian-Client-Patient Relationship (VCPR)**		
⬩ Unknown	38	15.7
⬩ No	114	47.1
⬩ Yes	90	37.1
✓ Cluster 1: Backyard farm for breeding (n = 115)	43	37.4
✓ Cluster 2: Backyard farm not for breeding (n = 75)	19	25.3
✓ Cluster 3: Small-scale farms (n = 52)	28	53.8

^#^ This question allowed respondent to select multiple responses

Multivariable logistic regression found that demographic factors were statistically significantly associated with responses about purchase and use of antibiotics in livestock/poultry in the past year prior to the survey [Table 4]. The odds of purchasing antibiotics in the last year was 1.4 to 2.2 times higher if they raised non-livestock/exotic pets (Odds ratio [OR] = 1.45); or raised equids (OR = 1.90), small ruminants (OR = 2.07) or livestock/poultry for sale (OR = 2.21), compared to those who did not raise those specific species/use. On the other hand, farmers who raised duck/geese/turkey (OR = 0.58) were less likely to purchase antibiotics for their livestock/poultry in the past year. The odds of using antibiotics was 1.6 to 2.4 times higher if farmers raised non-livestock/exotic pets (OR = 1.62), livestock/poultry for sale (OR = 1.97), pet/hobby/rescue (OR = 2.23), equids (OR = 2.40), or raised small ruminants (OR = 2.4), compared to those who did not raise those specific species/use. On the other hand, respondents who raised duck/geese/turkey (OR = 0.54) were less likely to use antibiotics in the past year compared to those who did not own these species.

**Table 4:** Results of the multivariable logistic regression models about antibiotics purchase and use in the past year prior to the survey.

Demographic factors associated with the purchase or the use of antibiotics in the past year prior to the survey	Odds Ratio (OR) [95% CI]	
	Antibiotics purchase	Antibiotics use
**Backyard producer for Sale**		
No	Reference	Reference
Yes	2.21 [1.15, 4.25]	1.97 [1.00, 3.89]
**Raise for Pet/Hobby/Rescue**		
No		Reference
Yes		2.23 [1.17, 4.30]
**Raise Duck, Turkey and/or Geese**		
No	Reference	Reference
Yes	0.58 [0.37, 0.91]	0.54 [0.34, 0.87]
Raise Small ruminants*		
No	Reference	Reference
Yes	2.07 [1.55, 2.84]	2.43 [1.77, 3.47]
Raise Equids**		
No	Reference	Reference
Yes	1.90 [1.06, 3.50]	2.40 [1.27, 4.70]
Raise Non-livestock / Exotic Pets***		
No	Reference	Reference
Yes	1.45 [1.02, 2.14]	1.62 [1.10, 2.51]

* Small ruminants: Goat, Sheep, Alpaca or Llama

** Equids: Horses, Mules or Donkeys

*** Non-livestock/Exotic Pets: Rabbit, Dog, Bee, Cavy, Turtle, Emu, Guinea fowls, Peacock, Pigeon, or Quail

### Impact of VFD and SB27 on the antibiotic use in livestock/poultry

Around one-fifth of respondents answered that VFD affected their decision on antibiotic use, and the proportion was similar among the three clusters of backyard and small-scale farms [Table 5]. The major changes after VFD implementation were more frequent veterinarian visits, fewer treated animals with antibiotics than before, and cessation of use of antibiotics in feed and water.

**Table 5:** Summary of the impact of veterinary feed directive (VFD) and SB27 on the antibiotic use.

Responses	Number	Proportion (%)
**The impact of VFD on the antibiotic use in practice**		
⬩ Unknown	49	20.2
⬩ No	151	62.4
⬩ Yes	42	17.4
✓ Cluster 1: Backyard farm for breeding (n = 115)	17	14.8
✓ Cluster 2: Backyard farm not for breeding (n = 75)	14	18.7
✓ Cluster 3: Small-scale farms (n = 52)	11	21.2
**How has the VFD affected your antibiotic use in practice** ^**#**^		
⬩ I have had to see my veterinarian more often	16	48.5
⬩ I treat fewer animals with antibiotics	14	42.4
⬩ I no longer use any antibiotics in feed	9	27.3
⬩ I no longer use any antibiotics in water	9	27.3
⬩ Increased difficulty to obtain antibiotics and additional cost	9	27.3
⬩ I have started keeping more complete records for my antibiotic use	7	21.2
⬩ I have needed to treat more individual animals	6	18.2
⬩ I now only use antibiotics in feed that do not require a VFD	6	18.2
⬩ I now only use antibiotics in water that do not require a prescription	3	9.1
**The impact of SB27 on the antibiotic use in practice**		
⬩ Unknown	53	21.9
⬩ No	124	51.2
⬩ Yes	65	26.9
✓ Cluster 1: Backyard farm for breeding (n = 115)	29	25.2
✓ Cluster 2: Backyard farm not for breeding (n = 75)	16	21.3
✓ Cluster 3: Small-scale farms (n = 52)	20	38.5
**How has SB27 affected your antibiotic use in practice** ^**#**^		
⬩ I have had to see my veterinarian more often	35	57.4
⬩ Increased difficulty to obtain antibiotics and additional cost	21	34.4
⬩ I treated fewer animals with antibiotics	14	23.0
⬩ I no longer use any antibiotics	9	14.8
⬩ I have started keeping more complete records for my antibiotic use	8	13.1
⬩ I have needed to treat more individual animals	7	11.5

^#^ This question allowed respondent to select multiple responses

Around one-third of respondents reported that SB27 affected their decision on antibiotic use [Table 5]. More respondents in small-scale farms answered that SB 27 affected their decision-making about antibiotic use than those who were in backyard farms. The major changes after SB27 implementation were more frequent veterinarian visits, additional costs for antibiotic use, and less use of antibiotics in livestock/poultry.

Multivariable logistic regression identified statistically significant demographic management factors associated with antibiotic use changes in livestock/poultry after the VFD and SB27 implementation [Table 6]. After the VFD implementation, 4-H/FFA members (OR = 3.53), farmers who purchased antibiotics (OR = 2.92) or used antibiotics in feed (OR = 3.26), were more likely to be impacted by VFD as compared to those who were not 4-H/FFA members or did not purchase or use antibiotics. After SB27 implementation, owners with a VCPR (OR = 2.82), made a decision of antibiotic use by themselves (OR = 4.39), or purchased antibiotics online (OR = 13.31) were more likely to be impacted by SB27.

**Table 6:** Results of the multivariable logistic regression models about the impact of veterinary feed directive (VFD) and Senate Bill 27 (SB27) on the use of antibiotics.

Factors associated with the impact of VFD or SB27 on the use of antibiotics	Odds Ratio (OR) [95% CI]	
	The impact of VFD on antibiotics use	The impact of SB27 on antibiotics use
4-H* or FFA** member		
No	Reference	
Yes	3.53 [1.67, 7.77]	
**Have a Veterinary-Client-Patient-Relationship (VCPR)**		
No		Reference
Yes		2.82 [1.42, 5.73]
**Antibiotic purchase in the past year**		
No	Reference	
Yes	2.92 [1.37, 6.40]	
**Antibiotic use in feed**		
No	Reference	
Yes	3.26 [1.14, 8.35]	
**Antibiotic purchase online**		
No		Reference
Yes		13.31 [2.10, 261.81]
**Antibiotic uses decided by themselves without any prescription**		
No		Reference
Yes		4.39 [1.88, 10.63]

*4-H is a US youth program housed by land-grant universities’ Cooperative Extension. This program provides developmental opportunities to youth (age 5 to 19) in rural farming communities, urban neighborhoods, and schoolyards. Skills learned include agriculture, animal husbandry, community service and personal development.

** FFA: National FFA Organization (formerly Future Farmers of America) is a US intracurricular student organization. It gives students experience in agriculture and leadership (middle and high school classes).

## Discussion

Our study investigated farm management of backyard and small-scale premises in California from 2018 to 2019, specifically looking at demographics, disease prevention and biosecurity procedures including antibiotic use and veterinary service, and the impact of two current pieces of legislation, VFD and SB27, on antibiotic use in these types of farms. We identified different antibiotic, preventive, and biosecurity practices that depended on farm demographics between backyard and small-scale farmers.

Most Californian backyard farmers generally raised a small number of chickens and/or small ruminants for personal use. They were classified into one of two clusters by their farm demographics. The first cluster of backyard farms raised poultry or livestock as pets (or hobby/rescue) for breeding purposes but the second cluster of backyard farms did not. Backyard farmers in the two clusters had similar herd/flock size and raised livestock/poultry mainly for personal use. If the backyard farmers also worked as a breeder, they used antibiotics more than those who did not. Considering that many backyard farmers used antibiotics limitedly for individual treatment of sick animals, antibiotics were used for the treatment of sick pets or breeding animals more than for production of animal products. Previous research on urban poultry/livestock reported that, even when backyard farmers raised animals mainly to obtain fresh animal products (meat, eggs, and milk), they still considered their livestock/poultry as pets [9, 23]. In addition, many backyard farmers raising animals for a breeding purpose (Cluster 1) were 4H/FFA members. Therefore, future outreach efforts targeting 4H/FFA programs would benefit from including topics related to baseline knowledge of judicious antibiotic use in sick animals, early disease diagnosis, and disease prevention in backyard livestock and poultry.

Around one-fifth of respondents were “small-scale commercial farmers” who raised fairly large numbers of livestock/poultry for sale and had different farm management practices compared to backyard farmers. Although the size of small-scale farms may be considered much smaller than large conventional commercial livestock farms, they tended to have a valid VCPR and use more antibiotics than the backyard farmers.

In the US, livestock farmers are no longer allowed to use antibiotics in feed and water of food-production animals for growth promotion and feed efficiency purposes, as of the VFD implementation in January 2017 [19]. In the present study, Californian backyard farmers reported very limited antibiotic use in feed (< 6%) or water (< 13%). California backyard and small-scale farmers reported that they used antibiotics mostly for the individual treatment of sick animals under veterinary oversight, with limited use for preventive purposes, unless animals had infectious digestive or respiratory diseases. These findings are similar to studies conducted in the UK and conventional cattle farms in the US. These cattle and sheep farmers reported low use of antibiotics, not for preventive purposes, but limitedly for individual treatments of significant diseases such as lameness, abortion, and neonatal diseases [24–27]. Although infrequent antibiotic use might decrease the potential risk of AMR emergence, low biosecurity practices and a lack of knowledge on prevention and control of diseases in backyard and small-scale farms potentially pose a risk for introduction of emerging and zoonotic disease spread in livestock of conventional farms [2, 4, 28]. Our study found that more than 35% of the respondents did not use veterinary services in the past year, and about 65% of respondents did not have a valid VCPR. Considering the current popularity of backyard poultry in the US, a lack of knowledge on disease prevention and control procedures in these biosecurity settings may increase the potential risk of zoonotic diseases infection (e.g., Salmonellosis) or the spread of avian infectious pathogens to commercial poultry farms (e.g., Avian Influenza and Newcastle Disease) [12, 15, 16, 29–31]. Therefore, proper guidance and education about disease prevention and biosecurity, simultaneous with veterinary oversight and antimicrobial stewardship, are required to promote better husbandry practices and animal health in backyard and small-scale farms, and reduce the risk of zoonotic and infectious disease spillover among conventional and backyard farm settings.

Our survey found that VFD and SB 27 significantly influenced farmers’ decisions on antibiotic use in Californian small-scale and backyard farms. In our study, only one-fifth of respondents reported that VFD affected their farm management in terms of disease prevention and biosecurity procedures (e.g., veterinary service and antibiotic use) both in backyard and small-scale farms, which was much lower than on conventional cattle farms [25, 26]. Californian backyard and small-scale farmers might be more likely to treat an individual animal with injectable antibiotics as compared to oral forms, given the challenge to acquire a VFD in the early implementation stages of this rule. Our survey also found that outreach efforts about on-farm management targeting 4H/FFA curricula would improve VFD compliance and promote disease prevention and biosecurity practices in backyard and small-scale farms. SB27 is a California regulation that requires a veterinary prescription for antibiotic purchase, traditionally brought over-the-counter, for therapeutic purposes only [20, 32]. In the present survey, respondents answered that SB27 (17.4%) and VFD (26.9%) affected their disease prevention and biosecurity procedures, specifically, more in the case of small-scale farmers as compared to backyard farmers. Therefore, we expected that, in practice, SB27 strongly encouraged backyard and small-scale farmers to have enhanced relationships with veterinarians through the VCPR and to have limited their antibiotic purchases online without veterinary oversight.

The veterinarian was a primary decision-maker for the purchase and use of antibiotics on Californian backyard and small-scale farms. Our survey found that more than 70% of respondents treated livestock with antibiotics under veterinarian oversight, which was higher than other US states reported in a previous study [29]. Specifically, more than half of small-scale farms had a VCPR, and nearly 70% of small-scale farms used veterinary services. Despite the importance of veterinarians for antimicrobial stewardship through the implementation of SB27 and VFD, backyard farmers in California still had limited access to veterinary services [4]. The present study identified a different level of access to veterinary oversight and a VCPR between small-scale and backyard farmers in California. More than two-thirds of backyard farmers did not have a VCPR and more than half of backyard farmers without the purpose of breeding (Cluster 2) did not use veterinary services in the past year prior to the survey, which was much higher than that of small-scale farms (Cluster 3, 28.8%). Furthermore, respondents generally sought veterinary service for a routine visit (59.6%) or emergency call (61.8%) but not frequently to discuss antibiotic use (e.g. VFD, prescription) (< 2%). Around half of respondents still used antibiotics on animals by their own decision (52.9%) and purchased directly from feed stores (36.5%) or online shops without the oversight of a veterinarian (14.6%). Many livestock farmers agreed with the effort of veterinarians to reduce the use of antimicrobials of public health importance in food-production animals worldwide [10, 24, 33, 34]. However, there were many barriers limiting the access to veterinary service for backyard farmers, such as the additional cost of veterinary care, small number of animals, additional recording-keeping and time required, and distance to veterinary service who was highly trained on livestock species in a backyard farm (e.g., backyard chicken and small ruminants) [4, 25, 27, 32]. Considering those barriers for backyard farmers to access veterinary services, additional programs of veterinary care are needed to reduce antimicrobial use and the potential risk of AMR in animal products intended for human consumption, such as promoting access to diagnostic laboratory services, online farm management software, or a stipend for additional costs for veterinary services [27, 35]. Furthermore, veterinary education should support the training of veterinarians who can provide adequate veterinary service on backyard farms [36, 37].

The first limitation of our study was the potential of geographical bias in Northern California. Although our survey broadly recruited backyard and small-scale farmers across many regions in California (46/58 counties), the number of participants in Northern California and the Northern San Joaquin Valley was much higher than that of Greater Southern California. California has very diverse micro-climate, geographical and environmental characteristics, and the farm management of backyard and small-scale farms should be adapted to the environmental conditions. Consequently, the large number of participants from Northern California and Northern San Joaquin valley may overrepresent the characteristics of farm management of backyard and small-scale farms of Northern California and underrepresent that of many urban backyard farms in Greater Southern California such as Los Angeles County [2]. Future studies should focus more on small-scale and backyard farms in Southern California. However, we still believe that our survey is informative because the herd/flock size, predominant species, and biosecurity levels of urban backyard farms in Southern California were not very different from those in Northern California [4, 23].

The second limitation was that our survey did not investigate a specific type and dose of antibiotic used on backyard and small-scale farms. The US livestock industry uses a variety of antibiotics for therapeutic purposes, and antimicrobial-resistant bacteria isolated from livestock reflect different antibiotic use profiles [27]. Previous studies addressed that the regulation with simple directions to reduce the amount of antibiotic use in the livestock industry might not be effective to prevent and control the emergence of AMR microorganisms because the livestock industry has used small amounts of antibiotics, limitedly [24, 25, 38]. Instead of the simple direction, the promotion of appropriate prescription by the veterinarian with detailed guidance and stewardship will be more effective to minimize indiscriminate antimicrobial use and reduce the risk of emergence of AMR microorganisms in food-producing animals [24]. Therefore, future research should investigate the antibiotic resistance profiles with the type and dose of antibiotics used on backyard and small-scale farms to specifically target recommendations to limit the emergence of AMR microorganisms in livestock and poultry on these types of premises.

## Conclusions

Backyard and small-scale livestock farming have recently expanded following the high demand for healthy and local livestock products in the US. The present study found that Californian backyard and small-scale farms generally raised chicken or small ruminants with small herd sizes for their personal use. Backyard and small-scale farmers mainly used antibiotics for individual treatment of livestock with the guidance of a veterinarian and rarely used antibiotics in feed or water. VFD and SB27 promoted appropriate use of antibiotics in livestock on California backyard and small-scale farms under veterinary oversight. However, many backyard farmers still had difficulty getting access to veterinary services. Therefore, considering the increase of popularity of backyard farming throughout the US, better access to veterinary services would help backyard and small-scale farmers to improve their knowledge of the importance of good husbandry practices with judicious antimicrobial use in livestock and finally contribute to reducing the risk of AMR in California as well as other states.

## Supporting information

S1 Table Description of survey questions, answers and variables. (DOCX)

S2 Table De-identified survey response data (n = 242). (XLSX)
