# Supplementary material for: Assessment of the knowledge and behavior of backyard and small-scale producers in California regarding disease prevention, biosecurity practices and antibiotics use
Source: PLoS One. 2022 Nov 21;17(11):e0277897. doi: 10.1371/journal.pone.0277897 (PMC9678316; doi:10.1371/journal.pone.0277897)
Supplement: S1 Table — (DOCX) [file pone.0277897.s001.docx]

S1 Table. The description of survey questions, answers and variables

| Number | Questions & Answers | | Variables & Values | |
| --- | --- | --- | --- | --- |
| 1 | **In which California county is your backyard premise/farm based?** | | **Participants’ premises by countries [Figure 1]** | |
| 2 | **Where is your property located?**   1. Urban (city > 250,000 inhabitants) 2. Town (city < 250,00 inhabitants and surrounded by a metropolitan area) 3. Suburban (periphery of metropolitan area and close to agricultural land or other open space) 4. Rural 5. Others* | | **Location of farms**  (1) Rural  (2) Town  (3) Suburban  (4) Urban | |
| 3 | **How would you describe the reason(s) why you raise livestock/poultry? ****  (1) 4-H or FFA member  (2) Pets  (3) Backyard producer for personal use  (4) Backyard producer for sale of live animals  (5) Backyard producer for sale of animal products (e.g., eggs, fiber, meat, etc.)  (6) Small-scale farmer (livestock only)  (7) Small-scale diversified farmer (vegetables and livestock)  (8) Breeder  (9) Hobby/Rescue  (10) Other (specify)  (a) School garden, Packing, Fairground, Experimental use | | **Reason to raise livestock/poultry**  (1) 4-H or FFA member  (2) Pets/Hobby/Rescue  (3) Backyard producer for personal use  (4) Backyard producer for sale  (4) Backyard producer for sale  (5) Small-scale farmer  (5) Small-scale farmer  (6) Breeder  (2) Pets/Hobby/Rescue  (7) Others | |
| 4-(1) | **What is the average size of your herd or flock? ****  (1) Chicken  (2) Ducks  (3) Turkeys  (4) Geese  (5) Goats  (6) Sheep  (7) Swine  (8) Beef Cattle  (9) Dairy Cattle  (10) Horses/Mules/Donkeys  (11) Other (specify)  (a) Rabbit, Bee, Cavy, Turtle, Emu, Guinea fowls, Peacock, Quail  (b) Alpaca, Llama | | **Species of livestock/poultry**  (1) Chicken  (2) Duck/Geese/Turkey  (2) Duck/Geese/Turkey  (2) Duck/Geese/Turkey  (3) Small Ruminants (Goats/ Sheep/Alpaca/Llama)  (3) Small Ruminants (Goats/ Sheep/Alpaca/Llama)  (4) Swine  (5) Cattle  (5) Cattle  (6) Equids (Horse/Mules/Donkeys)  (7) Non-Livestock / Exotic pets  (4) Small Ruminants (Goats/ Sheep/Alpaca/Llama) | |
| 4-(2) | **What is the average size of your herd or flock? ****  (1) 1-5 head  (2) 6-10 head  (3) 11-20 head  (4) 21-50 head  (5) 51-100 head  (6) >100 head  (7) No response | | **Herd/Flock size**  (1) 1-10 head  (1) 1-10 head  (2) 11-20 head  (3) 21-50 head  (4) > 51 head  (4) > 51 head  (5) 0 head | |
| 5 | **In the last 12 months, did you purchase antibiotics for your poultry or livestock?**  (1) Yes  (2) No | | **Purchase antibiotics in the past year prior to the survey**  (1) Yes  (2) No | |
| 5-(1) | **From where did you purchase your antibiotics? ****  (1) Directly from my veterinarian  (2) Mailed/delivered from a drug distributor with the order placed by a veterinarian  (3) Directly from feed store/retail store  (4) Directly from a drug distributor  (5) Online/internet  (6) Other (specify) * | | **Source of antibiobials purchase**  (1) Directly from a veterinarian  (2) Delivered of a drug distributor with the ordered by veterinarians  (3) Directly from feed store/retail store  (4) Directly from a drug distributor  (5) Online/internet | |
| 6 | **In the last 12 months, have you used antibiotics for treating livestock or poultry?**  (1) Yes  (2) No | | **Use antibiotics in the past year prior to the survey**  (1) Yes  (2) No | |
| 6-(1) | **Who decided that antibiotics were to be used for your backyard livestock or poultry?****  (1) Owner of farm/backyard owner (non-veterinarian)  (2) Family member (non-veterinarian)  (3) Employee (non-veterinarian)  (4) Veterinarian  (5) Service manager who oversees more than one operation (non-veterinarian)  (6) Other (specify) * | | **Decision of antibiotics use for livestock/poultry**  (1) Owner of farm/backyard owner (non-veterinarian)  (2) Family member (non-veterinarian)  (3) Employee (non-veterinarian)  (4) Veterinarian  (5) Service manager (non-veterinarian) | |
| 7 | **In the last 12 months, were your poultry or livestock given antibiotics in water?**  (1) Yes  (2) No | | **Use antibiotics in water in the past year prior to the survey**  (1) Yes  (2) No | |
| 8 | **In the last 12 months, were your poultry or livestock given antibiotics in feed?**  (1) Yes  (2) No | | **Use antibiotics in feed in the past year prior to the survey**  (1) Yes  (2) No | |
| 9 | **For each of the diseases or disorders listed below, please indicate under which circumstance you typically decide to treat your livestock or poultry with antibiotics. Please mark only one answer per disease/disorder.** | | **The circumstance to treat antibiotics to livestock/poultry by type of diseases/disorder of animals. [Figure 3]** | |
|  | (1) Respiratory disease  (2) Scours or other digestive disturbances/disease  (3) Lameness or other physical injury  (4) Eye problems  (5) Reproductive problems  (6) Mastitis  (7) Weak newborn/chicks or death of a sibling at birth  (8) Not eating  (9) Bad coat/feathers  (10) Other (specify) * | (1) I only treat the animal that is affected  (2) If one animal is affected, I treat it and the other animals in the group  (3) I only treat if multiple animals are affected  (4) I treat multiple animals before the condition arises  (5) I do not use antibiotics for this  (6) Does not apply | (1) Respiratory dz  (2) Digestive Dz  (3) Physical injury  (4) Eye problems  (5) Reproductive Dz  (6) Mastitis  (7) Weak newborn/sibling  (8) Not eating  (9) Bad coat/feathers  (10) Other (specify) * | (1) One sick, only sick animal  (2) One sick, treat the group  (3) Only treat multiple animals are sick  (4) Before sick, treat multiple animals.  (5) Do not use antibiotics  (6) Do nothing |
| 10 | **In the last 12 months, did you have a VCPR with a veterinarian or veterinary clinic for your animals?**  (1) Yes  (2) No  (3) I don’t know | | **Have a Veterinarian-Client-Patient Relationship (VCPR)**  (1) Yes  (2) No  (3) No response (Missing) | |
| 11 | **In the last 12 months did you use the services of a veterinarian (in-person, via phone, or by email)?**  (1) Yes  (2) No | | **Use of the services of a veterinarian in the past year prior to the survey**  (1) Yes  (2) No | |
| 11-(1) | **How did you use the services of a veterinarian? ****  (1) Regular or routine visits (ex: pregnancy checks, herd health visits, vaccinations, etc.)  (2) For certificates of veterinary inspection (ex: health certificates)  (3) Emergency calls (ex: birthing difficulty, multiple sick animals, sudden illness, etc.)  (4) Consulted over the phone or by email  (5) For feed VFDs and water prescriptions  (6) Other (specify) * | | **Decision of antibiotics use for livestock/poultry**  (1) Regular or routine visits  (2) For certificates of veterinary inspection  (3) Emergency calls  (4) Consulted over the phone or by email  (5) For feed VFDs and water prescriptions | |
| 12 | **On January 1, 2017, nation-wide laws went into effect that require a veterinary feed directive (VFD) to use antibiotics in feed or a prescription to use antibiotics in water. Did this legal change affect your antibiotic use practices?**  (1) Yes  (2) No  (3) I don’t know | | **The impact of VFD on the antibiotic use in practice**    (1) Yes  (2) No  (3) No response (Missing) | |
| 12-(1) | **How did this legal change regarding VFDs affect your antibiotic use practices?** ^**^  (1) I now only use antibiotics in feed that do not require a VFD  (2) I now only use antibiotics in water that do not require a prescription  (3) I no longer use any antibiotics in feed  (4) I no longer use any antibiotics in water  (5) I have had to see my veterinarian more often  (6) I have needed to treat more individual animals  (7) I treat fewer animals with antibiotics  (8) I have started keeping more complete records for my antibiotic use  (9) Other (specify)  (a) Increase difficulty to obtain antibiotics and additional cost  (10) Not applicable | | **How the VFD affect your antibiotic use in practices**  (1) I now only use antibiotics in feed that do not require a VFD  (2) I now only use antibiotics in water that do not require a prescription  (3) I no longer use any antibiotics in feed  (4) I no longer use any antibiotics in water  (5) I have had to see my veterinarian more often  (6) I have needed to treat more individual animals  (7) I treat fewer animals with antibiotics  (8) I have started keeping more complete records for my antibiotic use  (9) Increase difficulty to obtain antibiotics and additional cost  (10) No response (Missing) | |
| 13 | **On January 1, 2018, a California law (SB27) went into effect that requires a prescription to acquire/use antibiotics commonly sold in feed stores and by distributors. Did this legal change affect your antibiotic use practices?**  (1) Yes  (2) No  (3) I don’t know | | **The impact of SB27 on the antibiotic use in practice**    (1) Yes  (2) No  (3) No response (Missing) | |
| 13-(1) | **How did this legal change regarding SB27 affect your antibiotic use practices?** ^**^  (1) I no longer use any antibiotics  (2) I have had to see my veterinarian more often  (3) I have needed to treat more individual animals  (4) I treat fewer animals with antibiotics  (5) I have started keeping more complete records for my antibiotic use  (6) Other (specify)  (a) Increase difficulty to obtain antibiotics and additional cost  (7) Not applicable | | **How the VFD affect your antibiotic use in practices**  (1) I no longer use any antibiotics  (2) I have had to see my veterinarian more often  (3) I have needed to treat more individual animals  (4) I treat fewer animals with antibiotics  (5) I have started keeping more complete records for my antibiotic use  (6) Increase difficulty to obtain antibiotics and additional cost  (7) No response (Missing) | |

* These answers were not included as variable because no respondents selected

^**^ This question can select multiple responses
